# Recurrent endometrial atypical hyperplasia diagnosed by pathological examination of the placenta from a live birth: a case report

**DOI:** 10.1186/s12884-023-05972-0

**Published:** 2023-09-25

**Authors:** Weilu Wu, Wei Wang, Juan Zou

**Affiliations:** 1 Department of Pathology, West China Second University Hospital, Sichuan University, No. 20, Section 3, Renmin NanLu Chengdu, Chengdu, 610041 China; 2 Key Laboratory of Birth Defects and Related Diseases of Women and Children (Sichuan University), West China Second Hospital, Ministry of Education, Sichuan University, Chengdu, 610044 China

**Keywords:** Endometrial atypical hyperplasia, Placenta

## Abstract

**Background:**

Pregnancy complicated with endometrial atypical hyperplasia, which is often observed during early pregnancy, is extremely rare.

**Case presentation:**

The patient was a 30-year-old woman who had premature delivery at 30^+ 1^ weeks gestation, and endometrial atypical hyperplasia was discovered by placental examination.

**Conclusions:**

For patients who undergo fertility-sparing treatment for endometrial atypical hyperplasia, the evaluation of the decidua via the placental pathological examination is particularly important. These examinations make a great clinical contribution to the early detection and diagnosis of endometrial atypical hyperplasia.

**Supplementary Information:**

The online version contains supplementary material available at 10.1186/s12884-023-05972-0.

## Background

Progesterone therapy is the main fertility-sparing treatment for endometrial atypical hyperplasia and endometrial carcinoma. Pregnancy can be carried out after 6 months of fertility-sparing treatment with a pathological complete response [1–3]. Thus, endometrial evaluation during pregnancy and at delivery is the focus of long-term management after the treatment of endometrial atypical hyperplasia.

At present, the complete response rate of endometrial atypical hyperplasia after standard fertility-sparing treatment ranges from 62.9 to 81.1% [4–8], the recurrence rate ranges from 2.9 to 28.1% [8–10], and the pregnancy rate after complete response ranges from 18.8 to 83.3% [4–6, 8, 11–17]. The indications for post-pregnancy management are still unclear. There are no universally agreed-upon guidelines for this management. European Society of Gynaecological Oncology, the European Society for Radiotherapy & Oncology and the European Society of Pathology recommend definitive surgical treatment after the completion of childbearing, and patients who decline definitive surgery after delivery should be recommended to restart maintenance therapy with a levonorgestrel intrauterine device [18]. In China, multipoint biopsy of the decidua during caesarean delivery is recommend for patients who decline definitive surgery after delivery. Multidisciplinary consultation (MDT) decisions are made based on the biopsy evaluation results for patients whose endometrial evaluations show either progression or recurrence and who require fertility-sparing treatment or extra fascial hysterectomy [19–21]. Timely diagnosis after delivery can lead to early treatment. At present, there is no definite report on recurrence or progression shown by endometrial biopsy after delivery, and timely and comprehensive evaluation at delivery tends to be expected.

We report a case of recurrent endometrial atypical hyperplasia diagnosed at delivery, which was unexpectedly found through placental membrane histological examination. The purpose of this study was to provide a new noninvasive and comprehensive pathological method for the evaluation of post-pregnacy endometrium after fertility conservation therapy.

## Case presentation

**Fig. 1:**
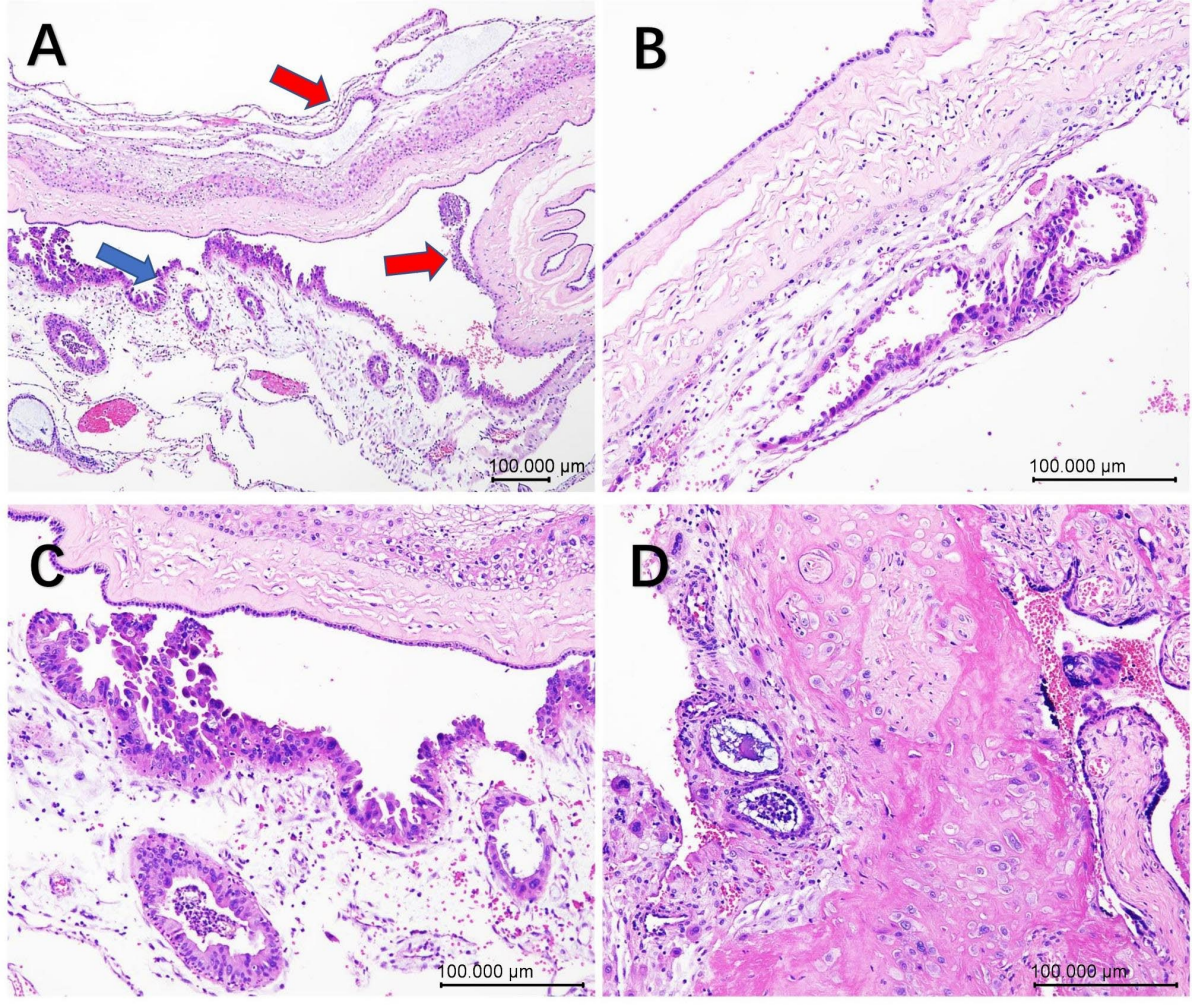
Histopathology of the decidua from the placental examination. **A**. Scanning view of the placental membrane shows that the capsular decidua and parietal decidua fused incompletely. The incompletely fused region of the decidua helps to distinguish the different parts of the decidua. The capsular decidua is marked by the red arrow, and the parietal decidua is marked by the blue arrow, x40. **B**. The capsular decidua with hyperplasia, x100. **C**. The parietal decidua with hyperplasia, x100. **D**. Atrophic basal decidua, x100

**Fig. 2:**
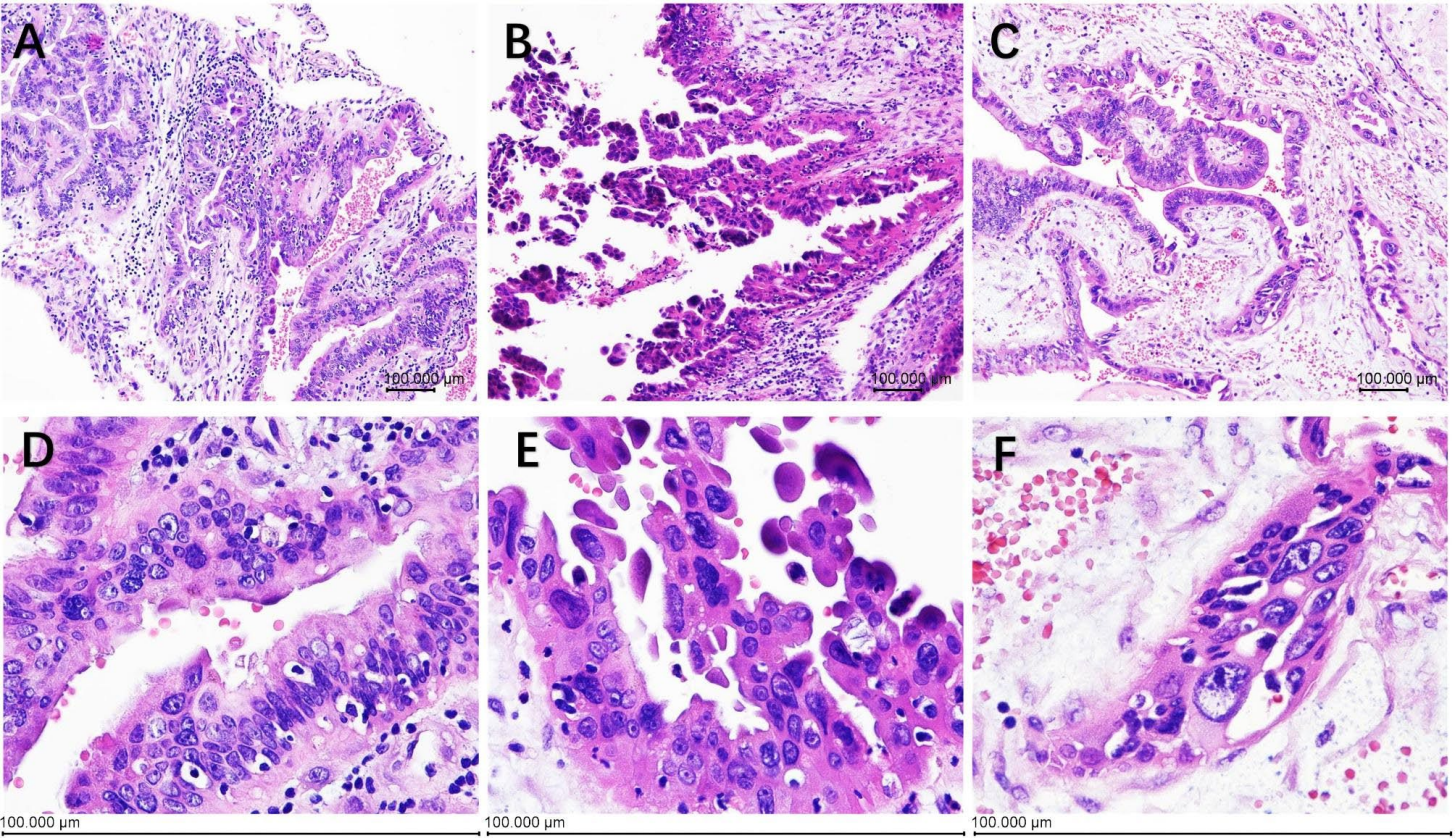
Endometrial atypical hyperplasia. (**A**-**C**) Scanning view of the architectural changes of the decidua, including glandular branching, dilating and papillary, x40. (**D**-**F**) High-power magnification showing the cellular atypia, characterized by loss of polarity, multiple stratified, clumped chromatin, and prominent nucleoli, x400

**Fig. 3:**
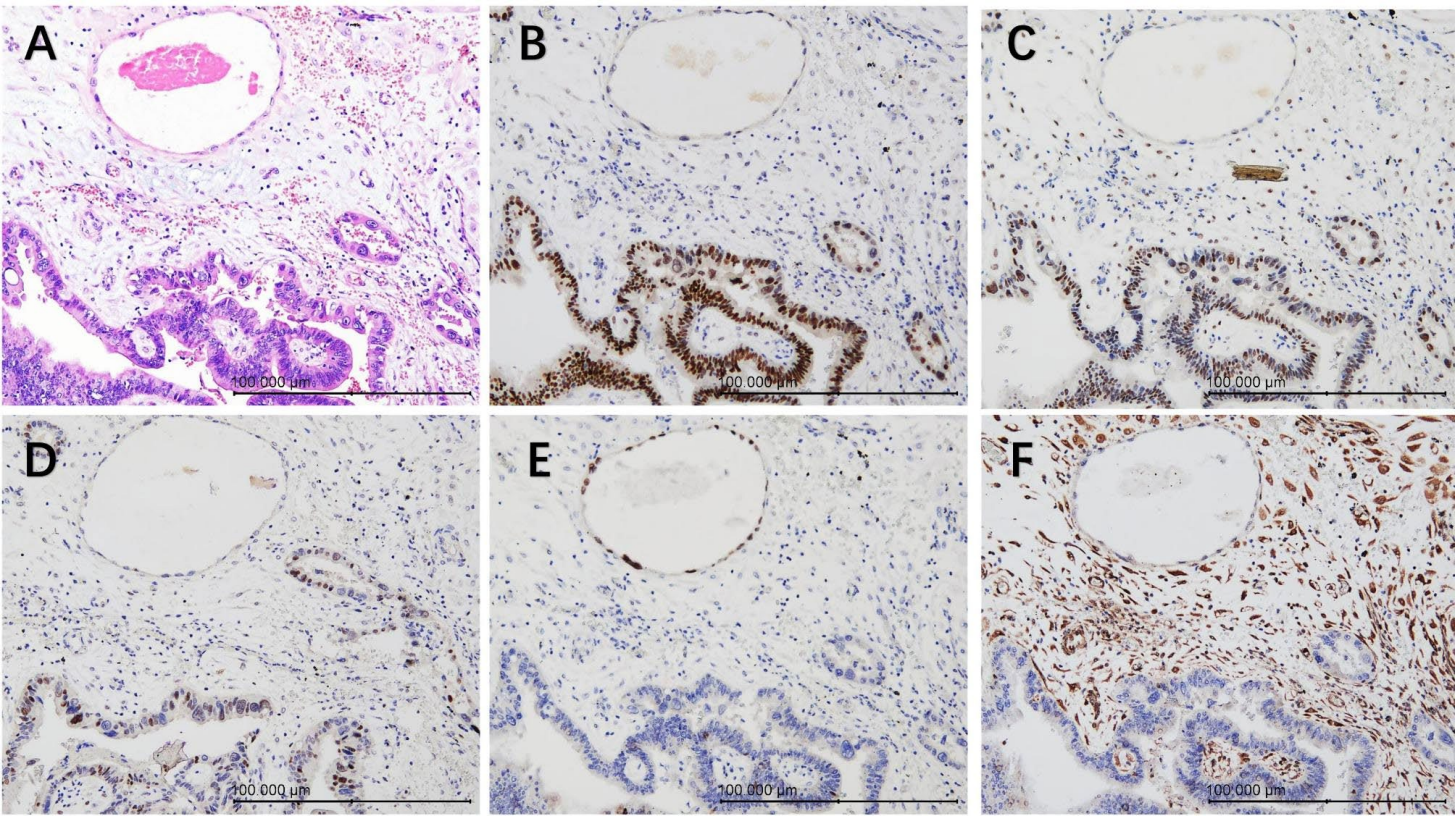
The atypical hyperplastic endometrium was distinct from the nonatypical gland. (**A**) The nuclear and cytoplasmic features of the branched glands below were distinct from the atrophic gland above, x200; (B-F) The atypical hyperplasia decidua epithelial cells were positive for ER (**B**) and PR (**C**) and negative for PTEN (**E**) and PAX2 (**F**), which was opposite to the atrophic gland, and with p53 wild type (**D**). x200

A 27-year-old G1P0 with known case of endometrial atypical hyperplasia presented with fertility-sparing treatment, pregnancy, and endometrial atypical hyperplasia recurrence. Four years ago, the patient underwent fertility-sparing treatment with megestrol acetate 160 mg Qd for 6 months, accompanied by hysteroscopic endometrial tissue biopsy every 3 months during the treatment. In the first 3 months, endometrial atypical hyperplasia was still observed by endometrial biopsy. A pathological complete response was obtained at the second 3 months biopsy (body mass index, BMI: 27.28 kg/m^2^). Unfortunately, 4 months later, endometrial atypical hyperplasia recurred and was diagnosed by hysteroscopy and pathological examination before in vitro fertilization (IVF), and a pathological complete response was obtained after fertility-sparing treatment with the Mirena IUD combined with metformin for 12 months (BMI: 23.50 kg/m^2^). The endometrium of the patient was assessed by transvaginal ultrasound monthly and endometrial tissue biopsy was performed every four months during this year. The first 4 months and second 4 months endometrial biopsies both showed that the endometrial glands were atrophic and that the endometrial stroma had decidual-like changes. In the last biopsy, the epithelium was positive for oestrogen receptor (ER) and negative for progesterone receptor (PR). Then, since the patient’s husband had developed asthenospermia, the patient underwent IVF. During the pregnancy, there was a small amount of vaginal bleeding with an unknown aetiology at 8 weeks of gestation. There were no other abnormalities observed in subsequent routine antenatal care until preterm delivery inevitably occurred at 30^+ 1^ weeks gestation due to cervical dysfunction and vaginal bleeding (BMI: 29.50 kg/m^2^). The newborn twins’ neonatal 1-, 5- and 10-min Apgar scores were 9-10-10 and 8-9-10, respectively, accompanied by meconium-stained amniotic fluid. Bedside ultrasound examination was routinely performed at delivery, and the placenta was sent for pathological examination. Ultrasonography indicated a slightly strong echo near the uterine fundus, with a maximum diameter of 2.9 cm. The pathological examination of the placenta showed a dichorionic-diamniotic unfused placenta measuring 21 cm x 16 cm x 2 and 17 cm x 11 cm x 1.5 cm, with complete and smooth foetal and maternal sides and no obvious abnormality of the umbilical cord or placental parenchyma. The placental membrane was translucent with a thickness of 0.1–0.2 cm, and the capsular decidua and parietal decidua had incompletely fused. Unexpectedly, the unfused epithelium showed multiple stratified and papillary hyperplasia, with moderate cellular atypia, an increased nucleoplasm ratio, clumped chromatin, and prominent nucleoli (Fig. 1). Moreover, there were some similar dilated branched hyperplasia glands scattered among the atrophic glands of the capsular decidua and parietal decidua (Fig. 2). The atypical hyperplastic epithelium was positive for ER and PR and negative for PTEN and PAX2, which was opposite to the surrounding atrophic epithelium (Fig. 3). A diagnosis of recurrent endometrial atypical hyperplasia was made.

The patient required follow-up for lactation, and postpartum ultrasonography, which was performed 3 months after delivery, showed that the endometrial thickness was 0.45 cm (single layer), the echo was uneven, the intrauterine strong echo had a punctured blood flow signal and persisted, and the maximum diameter was 0.4 cm. Therefore, hysteroscopy was performed, and the intrauterine shape was irregular. Multiple polypoid lesions were observed in the left uterine cornu of the uterus, and endometrial thickening was observed in the right cornu, left wall, and anterior and posterior wall of the uterus. Targeted biopsies and pathological examination showed focal endometrial atypical hyperplasia in the left cornu and left wall of the uterus. Upon the request of the patient and based on MDT, the patient was treated with the Mirena IUD for 6 months，and got a pathological complete response.

## Discussion

This is the first case of recurrent endometrial atypical hyperplasia diagnosed through the histological examination of a placental membrane from a live birth, which was determined by the pathological examination of placental tissue. This study provides a new method for endometrial evaluation at delivery and adds new content to the maternal disease spectrum of placental tissue examination indicators.

The reasons for the recurrence of endometrial dysplasia in this case were analysed. First, PR was not expressed in part of the glandular epithelium in the last endometrial biopsy performed before pregnancy. We speculated that the high progesterone levels during pregnancy could not inhibit the endometrial hyperplasia induced by elevated oestrogen levels, which is also consistent with literature reports that PR negativity is an independent risk factor for recurrence [22]. Second, this patient had high insulin levels, a BMI of 29.50 kg/m^2^ at delivery, less than 12 months of a single treatment, and no maintenance therapy, which were reported as possible factors affecting recurrence [3, 23–25]. Third, pregnancy may also have had an impact on the assessment of endometrial atypical hyperplasia recurrence in this patient. The foetus and placenta made it difficult to evaluate the endometrium in utero objectively and effectively. The gestation period was as long as 6 months, which was longer than the period of endometrial assessment performed every 6 months in nonpregnant patients, and the endometrial changes were not detected in time.

Interestingly, recurrent endometrial atypical hyperplasia in this patient was unexpectedly discovered by the examination of placental tissue at delivery. Intrauterine endometrial examination at the end of pregnancy after endometrial atypical hyperplasia treatment is recommended by the guidelines [1, 3]. However, there is no exact report of the examination of decidual tissue delivered with the placenta to diagnose endometrial atypical hyperplasia. In the literature, for patients who experienced relapse before pregnancy and a complete response from retreatment with fertility conservation therapy, the endometrial evaluation at delivery was as follows: Yijiao He reported that 5 patients had full-term pregnancies, and no abnormal endometrial biopsy results were obtained at delivery [26]. Park et al. evaluated 3 patients in South Korea, and the hysterectomy specimens were free of endometrial hyperplasia and endometrial cancer [27]. Endometrial cancer has been found accidentally during placental examination [28, 29]. The findings add a new dimension to the spectrum of maternal diseases in placental examination indicators [30].

The advantages of decidual tissue examination in the placenta are as follows: First, decidual tissue examination is suitable not only for caesarean section patients but also for patients with vaginal delivery. Patients with vaginal delivery do not have to wait 42 days after delivery for endometrial evaluation, which will shorten the evaluation cycle. Second, the decidua parietalis, the decidua capsularis and the decidua basalis in the placenta cover the entire uterine cavity, so the pathological examination of the endometrium can allow for an overall evaluation rather than just multipoint biopsy. Third, the placenta is an organ of pregnancy that is delivered naturally after delivery, so decidual tissue examination is a noninvasive procedure for the mothers. Therefore, evaluating the decidua via placental examination is particularly important, as it greatly contributes to the early detection and diagnosis of endometrial atypical hyperplasia.

The diagnosis of endometrial atypical hyperplasia via the placenta is also challenging. First, endometrial atypical hyperplasia should be distinguished from the reactive changes induced by elevated oestrogen and progesterone levels, such as metaplasia and the Arias-Stella reaction, which involve cellular changes. The immunohistochemical markers PAX2 and PTEN are useful for performing differential diagnosis, and mismatch repair can assist in the assessment of prognosis [1, 31, 32]. In addition, the amount of decidua attached to the focal placenta may be less than sufficient to make a diagnosis, and microscopic examination of the entire foetal membrane and decidua is needed to improve the diagnostic rate.

In conclusion, an increasing number of women are becoming pregnant after fertility-sparing hormonal treatment for endometrial hyperplasia disease. High levels of oestrogen and progestogen during pregnancy may induce secondary effects on the recovered endometrium, which may be nonspecific and asymptomatic. Pregnant women with a history of endometrial atypical hyperplasia are at risk of recurrent endometrial atypical hyperplasia. Especially for pregnant women with complications such as preterm birth or bleeding during pregnancy, the evaluation of endometrial lesions by the deciduae in the placenta at delivery is comprehensive and noninvasive, which is suitable for both caesarean section patients and patients with vaginal delivery.

### Electronic supplementary material

Below is the link to the electronic supplementary material.

Supplementary Material 1

## Data Availability

The datasets used and/or analysed during the current study are available from the corresponding author on reasonable request.
